# The Effects of Internet Exposure on Sexual Risk Behavior Among Sexually Experienced Male College Students in China: Cross-sectional Study

**DOI:** 10.2196/31847

**Published:** 2022-05-02

**Authors:** Junfang Xu, Yan Luo, Hengjin Dong, Gang Zhao

**Affiliations:** 1 School of Public Health Zhejiang University School of Medicine Hangzhou China; 2 Shenzhen Pingshan District Center for Disease Control and Prevention Hangzhou China; 3 Hangzhou Center for Disease Control and Prevention Hangzhou China

**Keywords:** college males, internet exposure, sexual partners, risk behavior, HIV, MSM, social networks, students, sexually transmitted infections, public health

## Abstract

**Background:**

As a young subgroup, college students have become the main users of mobile social networks. Considering that people can indiscriminately access explicit sexual content on the internet, coupled with the increase of HIV infections in male college students, the role of the internet in meeting sexual partners and its correlation to risky sexual behavior has become an important topic.

**Objective:**

The aim of this study is to explore the effects of internet exposure on sexual partners and sexual risk behavior among sexually experienced male college students.

**Methods:**

An institution-based cross-sectional study design was used to collect data through a paper-based questionnaire administered to male college students recruited from colleges and gay organizations in Hangzhou, Zhejiang Province, China. A total of 1045 sexually experienced male students were incorporated in our analysis, with the following information collected: sociodemographic characteristics, sexual intercourse–related behaviors, and sexually transmitted disease (STD) knowledge. Mann-Whitney *U* and Kruskal-Wallis tests were used to examine differences regarding basic characteristics and sexual risk behaviors between male college students who meet sexual partners via the internet and those who do not. Sequential logistic regression models were employed to examine the influence of meeting sexual partners via the internet on risky sexual behaviors after controlling for other factors.

**Results:**

The mean age of the sexually experienced male students was 21.6 (SD 2.0) years. The likelihood of risky sexual behavior was varied, yet it was the highest for those who aim to meet paid sexual partners (145/192, 75.5% to 19/22, 86.4%), followed by those seeking partners for love or romance (258/435, 59.3%). Compared to non-internet partner seekers, internet partner seekers tended to have more casual intercourse (292/542, 53.9% versus 51/503, 10.1%), paid intercourse (32/542, 5.9% versus 12/503, 2.4%), and intercourse with same-sex partners (349/542, 64.4% versus 41/503, 8.2%); they were also more likely to use psychoactive drugs (125/349, 35.8% versus 5/41, 12.2%) and have more than 2 partners. With the increase of HIV and STD knowledge, the probability of having unprotected intercourse decreased for non-internet partner seekers. However, it increased for internet partner seekers with a rising HIV knowledge score. Sequential logistic regression showed that meeting sexual partners on the internet was statistically associated with sexual risk behaviors with multiple sexual partners (odds ratio 4.434; *P*<.001).

**Conclusions:**

Meeting sexual partners via the internet is a common behavior among sexually experienced male college students, and those who meet partners on the internet exhibited higher levels of risky sexual behaviors although they had sufficient HIV and STD knowledge; this is especially true for students who aimed to find partners for sexual intercourse. Thus, more attention should be paid to young adults to address the risky sexual behaviors that may contribute to STD spread among this population.

## Introduction

With the development of information technology, China has entered the internet era. As of 2019, the number of internet users in China was 802 million, and there were up to 788 million mobile internet users [1]. The internet allows people with different cultures, from different regions to contact each other and can contain a diverse array of social beliefs, values, and subjects.

As a young subgroup, college students have become the main users of internet social networks. In 2016, a report on the internet behavior of Chinese teenagers released by the China Internet Network Information Center highlighted that young people aged 19-24 years accounted for the largest proportion of internet use, as high as 48.1% [2,3]. In addition, more than 90% of young people used mobile social networks, which far exceeded the overall level of internet users [2,3]. Moreover, meeting strangers has become one of the most popular mobile social functions for young college students [4,5]. For example, the “shake” mobile feature allows the user to add another user as a friend if they physically shake their mobile phones at the same time. With the characteristic of anonymity, the “drifting bottle” has also been popular among young students; it refers to the way that individuals can send drifting bottles to make friends without filling in real personal information. Another related mobile social function is called “find nearby people” and is based on the positioning of the user’s mobile phone, allowing them to find nearby people whom they can befriend. Moreover, the use of some dating social media platforms (ie, Momo and Tantan) was up to 20% among college students, and the number of paying users for Momo alone increased to 11.6 million people in 2018 [6,7]. Indeed, exposure to the internet has some benefits for young people, for example, finding answers to sexual health questions, which can help to avoid the embarrassment they may encounter by visiting health providers in person [8,9]. Moreover, the internet is a powerful resource for young people who are at an age when experiencing many health-related issues can result in feelings of confusion, loneliness, or embarrassment [8-10]. Thus, the knowledge they gain from the internet is an important tool for health promotion and solving health-related problems. However, exposure to the internet may have many negative effects on young people, especially college students. For example, having intercourse with partners one meets on the internet increases the risk of HIV infection.

Indeed, the rates of HIV infection among young people have increased while the incidence of HIV has decreased among the whole population. For example, young people ages 15-24 years were found to account for 32% of newly infected cases and the number of young people living with HIV and AIDS has increased by more than 480,000 in the last 20 years [11,12]. In China, more worryingly, the prevalence of new HIV infections among college students has increased significantly, with an annual growth rate of 30%-50% in recent years [13]. In 2017, the number of newly diagnosed students was 3077, a number that is 10 times higher than it was 10 years ago, and nearly 10 HIV infections per day were reported among college students, especially among male students [14].

With societal and technological development, we now live in a pluralistic society where individuals face a variety of behavior choices. A pluralistic society not only permits various ways of sexual satisfaction but also presents the threat of STDs, especially for college students who are sexually active [15]. The emergence of the internet within the pluralistic society has also allowed for various ways to prevent the transmission of STDs. Moreover, as elaborated by risk society theory, hazards can be caused by the environment (ie, the internet) as well as individual factors, such as knowledge, behavior choices, and personal characteristics. Therefore, it is urgent to understand the risky sexual behaviors of individuals (ie, college students) within some environments to reduce the risk of acquiring STDs within society.

To achieve the 90-90-90 goals toward HIV elimination by 2030, it is urgent to strengthen prevention efforts among this emerging high-risk population [16]. Although some studies have been conducted regarding the use of the internet and risky sexual behaviors, most of them focused on the general population of students and men who have sex with men (MSM) [17]. Little research has been done focusing on sexually experienced young male college students, who are sexually active and the key population to target for preventing HIV spread. Under this background, we aim to explore the use of the internet for meeting sexual partners among sexually experienced male college students to provide evidence for effective interventions to reduce the likelihood of risky sexual behaviors and prevent the spread of HIV among young college students.

## Methods

### Ethics Approval

Consent to participate was obtained from each participant before data collection. We did not collect any personally identifiable information. The study protocol and consent procedure were approved by the Medical Ethics Committee of the Hangzhou Center for Disease Control and Prevention (20190712).

### Participants

We used an institution-based cross-sectional study design to collect data from colleges and gay organizations located in Zhejiang Province, China. We chose these 2 sites for 2 reasons. First, the number of new HIV infections among college students has increased significantly in recent years. Second, this significant increase of new HIV infections has occurred mainly in male students as HIV disproportionately impacts MSM [14]. Therefore, we conducted the survey in the context of colleges and gay organizations to efficiently reach sexually experienced male students.

All college students who were studying in the 44 colleges between September 2020 and November 2020 were invited to participate in the investigation. The inclusion criteria for participants were (1) students who were studying in the 44 colleges and (2) male students. The exclusion criteria were (1) people who were not students studying in the colleges, (2) people under 18 years, (3) female students, (4) foreign students, and (5) students who did not want to participate in the investigation. However, only male students who had a sexual experience in the previous year were incorporated into the analysis, and these sexually experienced male students had experiences of ejaculation before. The sample size was calculated based on a 7.6% and 19% HIV diagnosis risk for men who did not meet sexual partners on the internet and men who did respectively [8] (α=.05), which requires at least 290 participants.




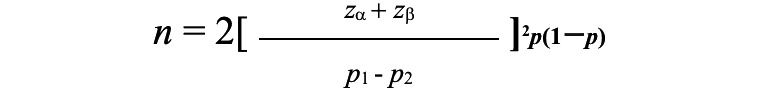




Finally, 1045 sexually experienced male students completed the questionnaire and were incorporated into our analysis.

### Data Collection

A paper-based questionnaire was used to collect related data among male college students; it was pretested and then revised based on the pretest. The following information was collected from the participants: sociodemographic characteristics (eg, age, education, residence, and years in school), sexual intercourse–related information (eg, sexual orientation, age at first sexual intercourse, and condom use), sexual intercourse–related behaviors in the past 6 months (eg, commercial, homosexual, and casual sexual intercourse and psychoactive drug use) and HIV and sexually transmitted disease (STD) knowledge. Years in school represents how many years it has been since a participant attended college. HIV and STD knowledge mainly represents college males’ understanding of the transmission routes and prevention methods for STDs such as HIV. HIV and STD knowledge was measured using the 18-item HIV Knowledge Questionnaire, which has been widely applied to HIV-related surveys in China and has been shown to have good validity [18], in addition to 4 questions measuring participants’ knowledge of other STDs, issued by the Hangzhou Center for Disease Control and Prevention. Responses were recorded as “true,” “false,” or “don’t know.” If the answer was correct, a score of 1 was assigned; a score of 0 was assigned if the answer was incorrect or a response of “don’t know” was provided. HIV and STD knowledge was measured by the total score, with a higher score indicating a higher level of HIV and STD knowledge.

### Data Analysis

Sociodemographic data from the male college students was analyzed using descriptive statistics with frequency and percentage. Risky sexual behavior was defined as having unprotected intercourse with 1 or more partners. Mann-Whitney *U* and Kruskal-Wallis tests were used to examine the differences regarding basic characteristics and risky sexual behaviors between men who meet sexual partners on the internet and those who do not. Moreover, sequential logistic regression models were employed to determine the independent influence of meeting sexual partners on the internet on unprotected intercourse with 1, 2, or more sexual partners (dependent variable) after controlling for other factors (eg, psychoactive drug use during intercourse, HIV knowledge, age, stage of study, years in school, field of study, residence, sexual orientation, and age at first intercourse). If the odds ratio (OR) is greater than 1, having the exposure increases the odds of engaging in the sexual risk behavior. The exposure decreases the odds of the sexual risk behavior if the OR is less than 1. All data analyses were completed using the statistical software SPSS (version 23.0; IBM Corp). Variables with *P*<.05 were considered statistically significant.

## Results

Table 1 shows the basic characteristics of male college students who had sexual experiences. The average age of male students who seek sexual partner(s) via the internet was 21.9 (SD 2.2) years, while it was 21.3 (SD 1.6) years for participants who do not seek sexual partners on the internet. Among men meeting partners on the internet, 73.6% (399/542) were studying for a bachelor’s degree. Regarding sexual orientation, 60% (325/542) and 19.2% (104/542) of men meeting partners on the internet were homosexual and bisexual, respectively, and most (426/503, 84.7%) who did not meet partners on the internet were heterosexual (*P*<.001). In addition, 62% (336/542) of participants meeting partners on the internet had sexual intercourse for the first time before the age of 18 years; only 43.7% (220/503) of those who did not meet partners on the internet had sexual intercourse before the age of 18 years (*P*<.001).

**Figure 1:**
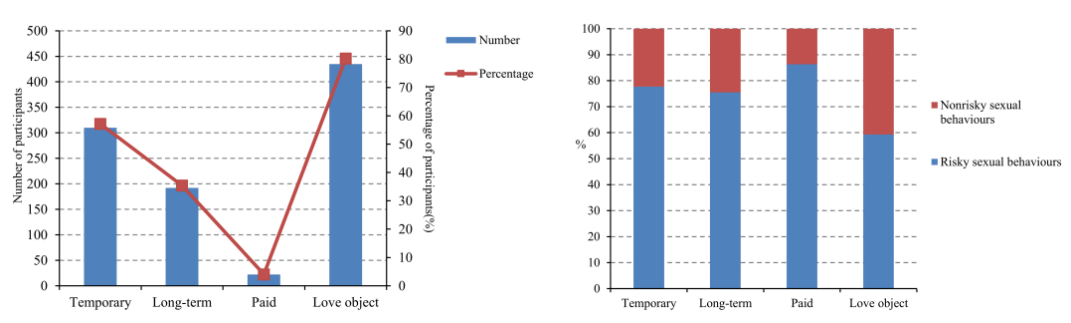
The aims for finding partners online (A) and the related risk sexual behaviors (B) for male college students.

Regarding the aims of finding partners on the internet (Figure 1), 80.3% (435/542) of participants aimed to find love or a romantic relationship, 57.2% (310/542) sought a temporary sexual relationship or one-time sexual intercourse, 35.4% (192/542) sought a stable partner for intercourse, and 4.1% (22/542) sought paid sexual intercourse. In addition, it was found that the likelihood of risky sexual behavior varied, and the risk was the highest for those seeking paid sexual partners (19/22, 86.4%), followed by those seeking casual sexual relationships (241/310, 77.7%), long-term sexual relationships (145/192, 75.5%), and romantic partners (258/435, 59.3%).

**Table 1:** Characteristics of male college students by internet exposure.

Characteristic		Value				*P* value
		Total participants (N=1045)	Participants who met sexual partners on the internet (n=542)	Participants who did not meet sexual partners on the internet (n=503)		
Age in years, mean (SD)		21.6 (2.0)	21.9 (2.2)	21.3 (1.6)	<.001	
**Stage of study, n (%)**						
	Professional training	95 (9.1)	59 (10.9)	36 (7.2)	.07	
	Bachelor	830 (79.4)	399 (73.6)	431 (85.7)		
	Master	100 (9.6)	69 (12.7)	31 (6.2)		
	PhD	20 (1.9)	15 (2.8)	5 (1)		
**Years in school, n (%)**						
	1	208 (22)	107 (19.7)	101 (20.1)	.007	
	2	308 (32.6)	144 (26.6)	164 (32.6)		
	3	228 (24.2)	109 (20.1)	119 (23.7)		
	4	176 (18.6)	114 (21.0)	62 (12.3)		
	5	18 (1.9)	13 (2.4)	5 (1)		
	≥6	6 (0.6)	4 (0.7)	2(0.4)		
	Missing^a^	101 (9.7)	51 (9.4)	50 (9.9)		
**Field of study, n (%)**						
	Non–health science	959 (91.8)	501 (92.4)	458 (91)	.42	
	Health science	86 (8.2)	41 (7.6)	45 (9)		
**Residence, n (%)**						
	Urban	770 (73.7)	385 (71)	385 (76.5)	.04	
	Rural	275 (26.3)	157 (29)	118 (23.5)		
**Sexual orientation, n (%)**						
	Heterosexual	539 (51.6)	113 (20.9)	426 (84.7)	<.001	
	Homosexual	372 (35.6)	325 (60)	47 (9.3)		
	Bisexual	134 (12.8)	104 (19.2)	30 (6)		
**Age at first sexual intercourse in years**						
	≤14	41 (3.9)	34 (6.3)	7 (1.4)	<.001	
	15-18	515 (49.3)	302 (55.7)	213 (42.4)		
	19-22	465 (44.5)	194 (35.8)	271 (53.9)		
	≥23	24 (2.3)	12 (2.2)	12 (2.4)		

^a^A total of 101 participants did not answer the questions regarding how many years of education they completed. Percentages are calculated based on the number of respondents who answered this category of questions.

Risky sexual behaviors of male college students who sought partners on the internet and offline are shown in Table 2. Most (292/542, 53.9%) of the participants who met partners on the internet had a casual sexual relationship, and 64.4% (349/542) of them had more than 2 casual partners; this was only 10.1% (51/503) for non-internet partner seekers, and most (38/503, 74.5%) of them only had 1 sexual partner (*P*<.001). In addition, 5.9% (32/542) of internet partner seekers and 2.4% (12/503) of offline partner seekers had paid sexual partners respectively (*P*=.005). Those who sought partners on the internet also tended to have intercourse with other men (349/542, 64.4% versus 41/503, 8.2%) and more than 2 same-sex partners (214/349, 61.3% versus 6/39, 14.7%) compared to non-internet partner seekers (*P*<.001). Moreover, they also tended to use psychoactive drugs during intercourse with same-sex partners (125/349, 35.8% for internet partner seekers versus 5/41, 12.2% for offline partner seekers; *P*=.003).

The rate of sexual risk behaviors among male students who seek sexual partners through websites on the internet was the highest (40/49, 81.6%), followed by those seeking partners through software platforms (361/475, 76%) and social media (133/217, 61.3%). The differences were statistically significant (*P*<.001).

**Figure 2:**
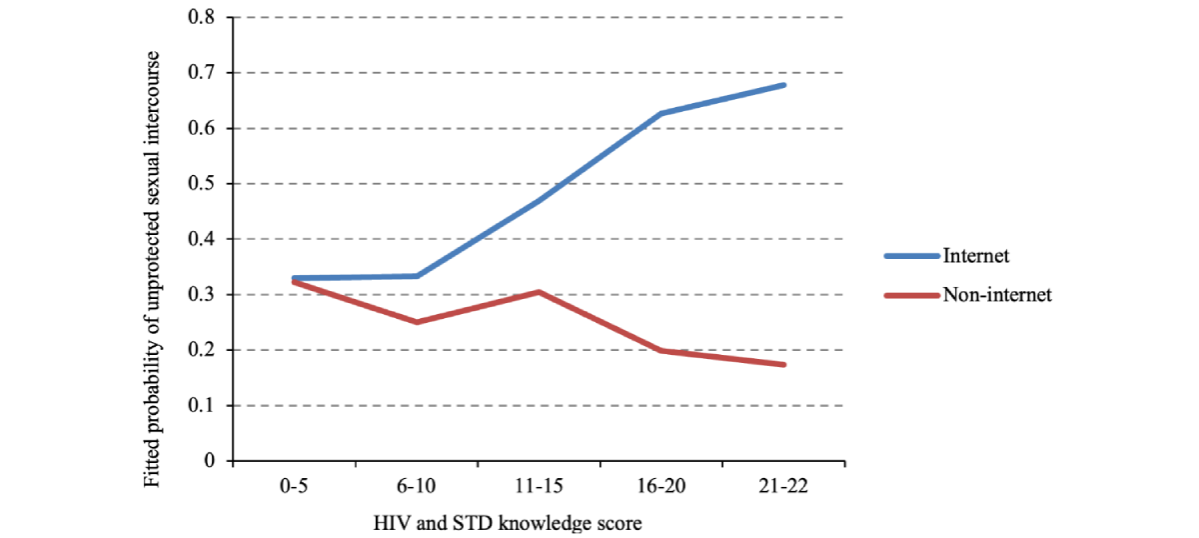
The fitted probability of engaging in unprotected sexual intercourse for male students who reported meeting sexual partners on the internet in the past 12 months and those who did not by HIV and sexually transmitted disease (STD) knowledge score.

The probability of engaging in unprotected intercourse after meeting partners on the internet or offline related to HIV knowledge level is shown in Figure 2. With the increase in HIV knowledge, the probability of having unprotected intercourse was lower for those who seek sexual partners offline. However, for those who seek sexual partners on the internet, the probability rose with the increase of the HIV knowledge score.

As shown in Multimedia Appendix 1, among the factors influencing unprotected intercourse with 1 or more partners among male college students, meeting sexual partners via the internet was statistically associated with engaging in risky sexual behaviors with multiple sexual partners (OR 4.434; *P*<.001). Moreover, those who did not use psychoactive drugs during intercourse were also found to have a low likelihood of sexual risk behaviors (OR 0.102; *P*<.001).

**Table 2:** Sexual risk behaviors of male college students by internet exposure.

Behavior				Total participants (N=1045), n (%)		Participants who met sexual partners on the internet (n=542), n (%)		Participants who did not meet sexual partners on the internet (n=503), n (%)		*P* value
**Protection at first intercourse**										
	Condom used			832 (79.6)		425 (78.4)		407 (80.9)		.32
	No condom used			213 (20.4)		117 (21.6)		96 (19.1)		
**Sexual experiences in the last 6 months**										
	**Ever had intercourse with casual partner**									
		Yes	343 (32.8)		292 (53.9)		51 (10.1)		<.001	
		No	702 (67.2)		250 (46.1)		452 (89.9)			
	**Number of casual partners^a^**									
		1	142 (41.4)		104 (35.6)		38 (74.5)		<.001	
		2	71 (20.7)		64 (21.9)		7 (13.7)			
		≥3	130 (37.9)		124 (42.5)		6 (11.8)			
	**Protection at last intercourse with casual partner^a^**									
		Condom used	308 (89.8)		264 (90.4)		44 (86.3)		.37	
		No condom used	35 (10.2)		28 (9.6)		7 (13.7)			
	**Ever had a paid sexual partner**									
		Yes	44 (4.2)		32 (5.9)		12 (2.4)		.005	
		No	1001 (95.8)		510 (94.1)		491 (97.6)			
	**Number of paid sexual partners^b^**									
		1	20 (45.5)		13 (40.6)		7 (58.3)		.37	
		2	14 (31.8)		11 (34.4)		3 (25.0)			
		≥3	10 (22.7)		8 (25.0)		2 (16.7)			
	**Protection at last intercourse with paid partner^b^**									
		Condom used	35 (81.4)		25 (78.1)		10 (83.3)		.81	
		No condom used	9 (20.9)		7 (21.9)		2 (16.7)			
	**Ever had a same-sex partner**									
		Yes	390 (37.3)		349 (64.4)		41 (8.2)		<.001	
		No	655 (62.7)		193 (35.6)		462 (91.8)			
	**Condom use during intercourse with same-sex partner^c^**									
		Never	18 (4.6)		16 (4.6)		2 (4.9)		.10	
		Sometimes	81 (20.8)		68 (19.5)		13 (31.7)			
		Always	291 (74.6)		265 (75.9)		26 (63.4)			
	**Number of same-sex partners^c^**									
		1	168 (43.3)		135 (38.7)		33 (80.5)		<.001	
		2	80 (20.6)		76 (21.8)		4 (9.8)			
		≥3	140 (36)		138 (39.5)		2 (4.9)			
	**Ever had intercourse with same-sex partner while using psychoactive drugs^c^**									
		Yes	130 (33.3)		125 (35.8)		5 (12.2)		.003	
		No	260 (66.7)		224 (64.2)		36 (87.8)			

^a^n is equal to the number of participants who ever had intercourse with a casual partner within each group of participants. Percentages are calculated accordingly.

^b^n is equal to the number of participants who ever had a paid sexual partner within each group of participants. Percentages are calculated accordingly.

^c^n is equal to the number of participants who ever had a same-sex partner within each group of participants. Percentages are calculated accordingly.

## Discussion

### Principal Findings

It is well known that the internet is a popular venue for MSM to seek sexual partners; for example, previous research has found that 40% of MSM used the internet to seek sexual partners [19-22]. However, besides this high-risk population, most college students also use the internet to find sexual partners [23], and this proportion was 51.9% (542/1045) among the sexually active male college students in our study. This is because nowadays, the universal use of smartphones and laptops allows young adults to spend more time in private to establish social relationships, including sexual relationships [24]. In addition, using the internet to find sexual partners is considered relatively anonymous and has a lower perceived risk of social rejection for sexually active people, especially those belonging to marginalized groups [25]. Indeed, in our study, we found that 79.2% (429/542) of students seeking a partner on the internet were homosexual or bisexual. Thus, it is not surprising that the free and generally nondiscriminatory space of the internet has become an appealing place for male college students to find sexual partners. In addition, compared to previous research which found that 33% of young people met sexual partners via the internet, our results showed a higher prevalence (542/1045, 51.9%) [26-29]. This may be because the participants in our study were sexually experienced male college students rather than the male students in general, which may lead to a higher proportion of participants using the internet to meet sexual partners. After analyzing web-based venues for meeting sexual partners using anonymous software, we found Momo, Tantan, Blued, and Aloha are the most commonly used (470/542, 86.7%) by male students with a high probability of risky sexual behaviors. This implies the importance of health promotion campaigns and interventions through these platforms, which are being used to find partners, especially for young students. Moreover, evidence has shown that integrating HIV prevention interventions into dating apps for MSM allows for the targeting of individuals who exhibit markers of risk in their profiles [30,31].

Men who reported meeting sexual partners on the internet also reported a higher frequency of psychoactive drug use and unprotected sexual intercourse compared with men who did not meet their partners on the internet. This result was consistent with previous studies [32]. But the high proportion of MSM among the sexually experienced males in our study may also lead to a high observed proportion of those who use psychoactive drugs. Indeed, evidence has also shown that up to 70% of MSM used psychoactive drugs [32]. Moreover, in our study, the association between meeting sexual partners on the internet and risky sexual behaviors remained significant even after adjusting for sociodemographic covariates and other HIV-related factors. However, it is unclear whether using the internet to meet sexual partners is a risk factor in itself or whether high-risk young adults tend to exhibit their risky sexual behaviors by meeting partners anonymously on the internet. But the internet is certainly an important venue for seeking partners for young adults; thus, awareness campaigns are urgent to target young college students who have recently met a new sexual partner on the internet.

We also found that risky sexual behaviors are more strongly associated with the purpose of finding a partner on the internet. For example, the likelihood of risky sexual behaviors was almost 30%-50% higher for those who find partners for sexual intercourse rather than romantic reasons. A study in Norway also suggested that associations between high-risk sexual behavior and seeking partners on the internet are more likely to be due to the individual’s aim for seeking partners using social media rather than all the related high-risk behaviors [33]. To maintain and promote the health, including reproductive health, of young people, it is also important to target high-risk sexual behaviors among male college students, especially bisexual male students who may transmit STDs to both men and women [34,35]. Further, HIV and STD knowledge was found to be an effective measure against risky sexual behavior [36]. However, the probability of risky sexual behaviors actually increased with a higher level of HIV and STD knowledge among male students who meet partners on the internet. This may be because although most young men are mindful of the risks of sexual behaviors [37,38], they may not always be aware of the consequences of their own risk-taking. In addition, sensation-seeking and a lack of impulse control among these young adults may also increase their likelihood of engaging in risky sexual behaviors [39]. Moreover, young people tend to focus on the benefits rather than the risks associated with engaging in sexual behaviors with partners they meet on the internet [40]; this leads to them feeling less vulnerable to the negative consequences associated with these behaviors. This may remind us that focusing on traditional education through knowledge inculcation (eg, teaching students about HIV transmission routes or risk factors for STDs) among male college students is not effective enough to reduce the likelihood of risky sexual behaviors, especially for students meeting partners on the internet. Instead, enhancing their awareness of the negative consequences of risky sexual behaviors may be effective. For example, this approach may involve communicating the negative effects of risky sexual behaviors on life, study, work, and social communication by interviewing HIV-positive students in the form of a video to deter students from engaging in high-risk sexual behaviors to some extent. To avoid the transmission of STDs by individuals with latent infections who do not know their HIV status, it is vital to increase HIV testing among high-risk college students. Furthermore, considering the continued high prevalence of risky sexual behaviors among people diagnosed with HIV, especially in low- and middle-income countries [41], it is urgent to increase surveillance of positive cases to reduce HIV transmission among college students. Simultaneously, treatment as prevention has been shown to be an effective way to reduce HIV incidence; thus, improving pretest and posttest counselling to promote adherence to highly active antiretroviral therapy is also important for HIV prevention [42].

### Conclusions

Our study suggests that most sexually experienced male college students engage in sexual behaviors with partners they meet on the internet. Those who met partners on the internet exhibited higher levels of risky sexual behaviors although they had sufficient HIV and STD knowledge, especially those who sought partners for intercourse. Therefore, it is urgent to offer support to help young male students better assess the risks of sexual behaviors on the internet, especially those who aim to find sexual partners. However, the traditional method of health education by spreading knowledge seems ineffective to reduce the likelihood of risky sexual behaviors, especially for students meeting partners on the internet. Instead, enhancing their awareness of the negative consequences (eg, conveying the negative effects on life) of risky sexual behaviors may be effective to deter these high-risk students to some extent. Moreover, it is vital to strengthen HIV testing among sexually experienced college students to avoid transmission by those with latent infections who do not know their HIV status.

### Limitations

Our study has some limitations. First, considering the cross-sectional study design, the data can only provide an indication of the association between sexual partner–seeking on the internet and high-risk sexual behaviors. The causal relationship between these behaviors cannot be decided; that is, it is unclear whether meeting sexual partners via the internet is a risk in itself or whether high-risk young adults tend to exhibit their risky sexual behaviors by meeting partners anonymously on the internet. Second, due to the social desirability bias, the participants may have underreported related risk behaviors. Third, considering the sample consisted of sexually experienced male students and the study was conducted in the settings of colleges and gay organizations, the results cannot be generalized to the overall population of college students. Moreover, given the significant variation in cultures, economies, and traditions across China, data from one province are unlikely to be nationally representative. Furthermore, future studies should pay more attention to the effects of possible endogenous variables influencing high-risk sexual behaviors.

## Appendix

Multimedia Appendix 1 Sequential logistic regression predicting risk sex with 1 or more partners among male college students.

## Abbreviations

MSM: men who have sex with men
OR: odds ratio
STD: sexually transmitted disease

## Data Availability

All the main data have been included in the results. Additional materials with details may be obtained from the corresponding author.

## References

[1] (2018). The 42nd China statistical report on internet development. China Internet Network Information Center.

[2] (2016). Research report on Chinese teenagers' online behavior in 2015. China Internet Network Information Center.

[3] Marazziti D, Baroni S, Mucci F, Piccinni A, Ghilardi A, Fiorillo A, Massimetti G, Luciano M, Sampogna G, Moroni I, Dell'Osso L (2020). Characteristics of internet use amongst Italian university students. Psychiatr Danub.

[4] Chan J, Ghose A (2014). Internet's dirty secret: assessing the impact of online intermediaries on HIV transmission. MIS Q.

[5] Choi EP, Wong JY, Lo HH, Wong W, Chio JH, Fong DY (2016). The impacts of using smartphone dating applications on sexual risk behaviours in college students in Hong Kong. PLoS One.

[6] Zhang QQ, Tang XL (2017). A survey of college students' use of mobile social software. Journal News Research.

[7] (2018). Momo second quarter 2018 financial results. The Paper.

[8] Cabecinha M, Mercer CH, Gravningen K, Aicken C, Jones KG, Tanton C, Wellings K, Sonnenberg P, Field N (2017). Finding sexual partners online: prevalence and associations with sexual behaviour, STI diagnoses and other sexual health outcomes in the British population. Sex Transm Infect.

[9] Aventin A, Gough A, McShane T, Gillespie K, O'Hare L, Young H, Lewis R, Warren E, Buckley K, Lohan M (2020). Engaging parents in digital sexual and reproductive health education: evidence from the JACK trial. Reprod Health.

[10] Bailey J, Mann S, Wayal S, Hunter R, Free C, Abraham C, Murray E (2015). Sexual Health Promotion for Young People Delivered via Digital Media: A Scoping Review.

[11] Joint United Nations Programme on HIV and AIDS (UNAIDS). UNAIDS.

[12] UNICEF. UNICEF.

[13] Li G, Jiang Y, Zhang L (2019). HIV upsurge in China's students. Science.

[14] Zou H, Tucker JD, Fan S, Xu J, Yu M, Luo Z, Cai W, Grulich AE (2018). Learning about HIV the hard way: HIV among Chinese MSM attending university. Lancet Infect Dis.

[15] Xu J (2021). Performance sex education and sexual health of contemporary college students. China Continuing Medical Education.

[16] Nguyen P, Gilmour S, Le P, Onishi K, Kato K, Nguyen H (2021). Progress toward HIV elimination goals: trends in and projections of annual HIV testing and condom use in Africa. AIDS.

[17] Khosropour CM, Johnson BA, Ricca AV, Sullivan PS (2013). Enhancing retention of an Internet-based cohort study of men who have sex with men (MSM) via text messaging: randomized controlled trial. J Med Internet Res.

[18] Wang Y, Jia M, Yuan D, Liang A, Zhang Z, Jiang X, Chen Y, Zhu H, Luo M, Wang Z, Cai Y (2019). Assessing consistent condom use among migrant men who have sex with men in Shanghai, China: validation of an information-motivation-behavioural skills model. BMC Infect Dis.

[19] Phillips G, Magnus M, Kuo I, Rawls A, Peterson J, Jia Y, Opoku J, Greenberg AE (2014). Use of geosocial networking (GSN) mobile phone applications to find men for sex by men who have sex with men (MSM) in Washington, DC. AIDS Behav.

[20] Holloway IW, Pulsipher CA, Gibbs J, Barman-Adhikari A, Rice E (2015). Network influences on the sexual risk behaviors of gay, bisexual and other men who have sex with men using geosocial networking applications. AIDS Behav.

[21] Weinrich JD (2015). Strange bedfellows: homosexuality, gay liberation, and the internet. J Sex Educ Ther.

[22] Goedel WC, Halkitis PN, Duncan DT (2016). Behavior- and partner-based HIV risk perception and sexual risk behaviors in men who have sex with men (MSM) who use geosocial-networking smartphone applications in New York City. J Urban Health.

[23] Döring Nicola, Daneback K, Shaughnessy K, Grov C, Byers ES (2017). Online sexual activity experiences among college students: a four-country comparison. Arch Sex Behav.

[24] Jonsson LS, Bladh M, Priebe G, Svedin CG (2015). Online sexual behaviours among Swedish youth: associations to background factors, behaviours and abuse. Eur Child Adolesc Psychiatry.

[25] Pachankis J, Hatzenbuehler M, Hickson F, Weatherburn Peter, Berg Rigmor C, Marcus Ulrich, Schmidt Axel J (2015). Hidden from health: structural stigma, sexual orientation concealment, and HIV across 38 countries in the European MSM Internet Survey. AIDS.

[26] Buhi E, Klinkenberger N, McFarlane M, Kachur R, Daley EM, Baldwin J, Blunt HD, Hughes S, Wheldon CW, Rietmeijer C (2013). Evaluating the internet as a sexually transmitted disease risk environment for teens: findings from the communication, health, and teens study. Sex Transm Dis.

[27] Daneback K, Månsson SA, Ross M (2007). Using the internet to find offline sex partners. Cyberpsychol Behav.

[28] Buhi E, Cook R, Marhefka S, Blunt HD, Wheldon C, Oberne AB, Mullins JC, Dagne GA (2012). Does the internet represent a sexual health risk environment for young people?. Sex Transm Dis.

[29] Young SD, Rice E (2011). Online social networking technologies, HIV knowledge, and sexual risk and testing behaviors among homeless youth. AIDS Behav.

[30] Pennise M, Inscho R, Herpin K, Owens J, Bedard BA, Weimer AC, Kennedy BS, Younge M (2015). Using smartphone apps in STD interviews to find sexual partners. Public Health Rep.

[31] Winetrobe H, Rice E, Bauermeister J, Petering R, Holloway IW (2014). Associations of unprotected anal intercourse with Grindr-met partners among Grindr-using young men who have sex with men in Los Angeles. AIDS Care.

[32] Zhao P, Tang S, Wang C, Zhang Y, Best J, Tangthanasup TM, Huang S, Yang B, Wei C, Tucker JD, Tang W (2017). Recreational drug use among Chinese MSM and transgender individuals: results from a national online cross-sectional study. PLoS One.

[33] Gravningen K, Aicken CR, Schirmer H, Mercer CH (2016). Meeting sexual partners online: associated sexual behaviour and prevalent chlamydia infection among adolescents in Norway: a cross-sectional study. Sex Transm Infect.

[34] Nguyen PT, Rahman MS, Le PM, Nguyen HV, Vu KD, Nguyen HL, Dao ATM, Khuong LQ, Hoang MV, Gilmour S (2021). Trends in, projections of, and inequalities in reproductive, maternal, newborn and child health service coverage in Vietnam 2000-2030: A Bayesian analysis at national and sub-national levels. Lancet Reg Health West Pac.

[35] Wu J, Wang L, Zhao G, Zhang X (2006). Sexual abuse and reproductive health among unmarried young women seeking abortion in China. Int J Gynaecol Obstet.

[36] Nachman S, Townsend CL, Abrams EJ, Archary M, Capparelli E, Clayden P, Lockman S, Jean-Philippe P, Mayer K, Mirochnick M, McKenzie-White J, Struble K, Watts H, Flexner C (2019). Long-acting or extended-release antiretroviral products for HIV treatment and prevention in infants, children, adolescents, and pregnant and breastfeeding women: knowledge gaps and research priorities. Lancet HIV.

[37] Bolding G, Davis M, Hart G, Sherr L, Elford J (2006). Heterosexual men and women who seek sex through the internet. Int J STD AIDS.

[38] Daneback K, Månsson SA, Ross MW (2007). Using the internet to find offline sex partners. Cyberpsychol Behav.

[39] Sonnenberg P, Clifton S, Beddows S, Field N, Soldan K, Tanton C, Mercer CH, da Silva FC, Alexander S, Copas AJ, Phelps A, Erens B, Prah P, Macdowall W, Wellings K, Ison CA, Johnson AM (2013). Prevalence, risk factors, and uptake of interventions for sexually transmitted infections in Britain: findings from the National Surveys of Sexual Attitudes and Lifestyles (Natsal). Lancet.

[40] Office for National Statistics (2010). Standard Occupational Classification 2010.

[41] Nguyen PT, Gilmour S, Le PM, Nguyen TT, Tanuma J, Nguyen HV (2021). Factors associated with high-risk behaviors of people newly diagnosed with HIV/AIDS: results from a cross-sectional study in Vietnam. AIDS Care.

[42] Le PM, Nguyen PT, Nguyen HV, Bui DH, Vo SH, Nguyen NV, Nguyen TV, Tran AT, Le AD, Ha NM, Dao AT, Gilmour S (2021). Adherence to highly active antiretroviral therapy among people living with HIV and associated high-risk behaviours and clinical characteristics: a cross-sectional survey in Vietnam. Int J STD AIDS.

