# Supplementary material for: The Effects of Internet Exposure on Sexual Risk Behavior Among Sexually Experienced Male College Students in China: Cross-sectional Study
Source: JMIR Public Health Surveill. 2022 May 2;8(5):e31847. doi: 10.2196/31847 (PMC9112083; doi:10.2196/31847)
Supplement: Multimedia Appendix 1 [file publichealth_v8i5e31847_app1.docx]

Multimedia Appendix 1**.** Sequential logistic regression predicting risk sex with one or more partner among male college students.

| **Items** |  | **Model 1** |  |  |  | **Model 2** |  |  |  | **Model 3** |  |  |  | **Model 4** |  |  |
| --- | --- | --- | --- | --- | --- | --- | --- | --- | --- | --- | --- | --- | --- | --- | --- | --- |
|  | Percentage  (%) | COR | 95%CI | P value |  | AOR | 95%CI | P value |  | AOR | 95%CI | P value |  | AOR | 95%CI | P value |
| **Step 1** |  |  |  |  |  |  |  |  |  |  |  |  |  |  |  |  |
| Met online sexual partner |  |  |  |  |  |  |  |  |  |  |  |  |  |  |  |  |
| Yes | 51.9 | 15.197 | 8.815,26.197 | <0.0001 |  | 5.547 | 3.040,10.122 | <0.0001 |  | 5.559 | 3.035,10.183 | <0.0001 |  | 4.434 | 2.268,8.870 | <0.0001 |
| No(Ref) | 48.1 |  | - | - |  | - | - | - |  |  |  |  |  | - | - | - |
| **Step 2** |  |  |  |  |  |  |  |  |  |  |  |  |  |  |  |  |
| Psychoactive drugs use during sex |  |  |  |  |  |  |  |  |  |  |  |  |  |  |  |  |
| No | 62.7 | 0.074 |  |  |  | 0.160 | 0.101,0.253 | <0.0001 |  | 0.170 | 0.107,0.269 | <0.0001 |  | 0.102 | 0.047,0.218 | <0.0001 |
| Yes(Ref) | 37.3 |  |  |  |  | - | - | - |  | - | - | - |  | - | - | - |
| **Step 3** |  |  |  |  |  |  |  |  |  |  |  |  |  |  |  |  |
| HIV/STDs knowledge |  |  |  |  |  |  |  |  |  |  |  |  |  |  |  |  |
| 0-5 | 1.8 | 0.040 |  |  |  |  |  |  |  | 0.146 | 0.003,7.206 | 0.334 |  | 0.127 | 0.002,8.436 | 0.335 |
| 6-10 | 1.1 | 0.325 |  |  |  |  |  |  |  | 0.630 | 0.062,6.434 | 0.630 |  | 0.559 | 0.054,5.749 | 0.625 |
| 11-15 | 5.3 | 0.325 |  |  |  |  |  |  |  | 0.375 | 0.136,1.033 | 0.375 |  | 0.423 | 0.144,1.248 | 0.119 |
| 16-20 | 60.5 | 0.619 |  |  |  |  |  |  |  | 0.677 | 0.464,0.988 | 0.677 |  | 0.589 | 0.383,0.904 | 0.016 |
| 21-22(Ref) | 31.4 |  |  |  |  |  |  |  |  | - | - | - |  | - | - | - |
| **Step 4** |  |  |  |  |  |  |  |  |  |  |  |  |  |  |  |  |
| Current age(continuous) | - | 1.134 |  |  |  |  |  |  |  |  |  |  |  | 1.159 | 0.993，1.353 | 0.062 |
| Age of first sex |  |  |  |  |  |  |  |  |  |  |  |  |  | - | - | - |
| ≤14 | 3.9 | 3.526 |  |  |  |  |  |  |  |  |  |  |  | 6.999 | 1.359,36.054 | 0.020 |
| 15-18 | 49.3 | 1.428 |  |  |  |  |  |  |  |  |  |  |  | 3.547 | 0.831,15.128 | 0.087 |
| 19-22 | 44.5 | 0.569 |  |  |  |  |  |  |  |  |  |  |  | 1.185 | 0.288,4.904 | 0.815 |
| ≥23(Ref) | 2.3 |  |  |  |  |  |  |  |  |  |  |  |  | - | - | - |
| Stages of study |  |  |  |  |  |  |  |  |  |  |  |  |  |  |  |  |
| Professional training | 9.1 | 0.623 |  |  |  |  |  |  |  |  |  |  |  | 0.149 | 0.288,7.783 | 0.635 |
| Bachelor | 79.4 | 0.460 |  |  |  |  |  |  |  |  |  |  |  | 1.048 | 0.224,4.901 | 0.953 |
| Master | 9.6 | 0.738 |  |  |  |  |  |  |  |  |  |  |  | 0.652 | 0.155,2.737 | 0.559 |
| PhD(Ref) | 1.9 |  |  |  |  |  |  |  |  |  |  |  |  | - | - | - |
| Years in school |  |  |  |  |  |  |  |  |  |  |  |  |  |  |  |  |
| 1 | 22.0 | 0.098 |  |  |  |  |  |  |  |  |  |  |  | 0.039 | 0.003,0.483 | 0.011 |
| 2 | 32.6 | 0.089 |  |  |  |  |  |  |  |  |  |  |  | 0.035 | 0.003,0.434 | 0.009 |
| 3 | 24.2 | 0.077 |  |  |  |  |  |  |  |  |  |  |  | 0.035 | 0.003,0.444 | 0.010 |
| 4 | 18.6 | 0.193 |  |  |  |  |  |  |  |  |  |  |  | 0.058 | 0.005,0.737 | 0.028 |
| 5 | 1.9 | 0.250 |  |  |  |  |  |  |  |  |  |  |  | 0.075 | 0.005,1.138 | 0.062 |
| ≥6(Ref) | 0.6 |  |  |  |  |  |  |  |  |  |  |  |  | - | - | - |
| Field of study |  |  |  |  |  |  |  |  |  |  |  |  |  |  |  |  |
| Health science | 8.2 | 0.660 |  |  |  |  |  |  |  |  |  |  |  | 0.558 | 0.247,1.252 | 0.158 |
| Non health science(Ref) | 91.8 |  |  |  |  |  |  |  |  |  |  |  |  | - | - | - |
| Residence |  |  |  |  |  |  |  |  |  |  |  |  |  |  |  |  |
| Urban | 73.7 | 0.989 |  |  |  |  |  |  |  |  |  |  |  | 1.477 | 0.928,2.352 | 0.100 |
| Rural(Ref) | 26.3 |  |  |  |  |  |  |  |  |  |  |  |  | - | - | - |
| Sexual orientation |  |  |  |  |  |  |  |  |  |  |  |  |  |  |  |  |
| Heterosexual(Ref) | 51.6 |  |  |  |  |  |  |  |  |  |  |  |  | - | - | - |
| MSM | 35.6 | 10.533 |  |  |  |  |  |  |  |  |  |  |  | 0.651 | 0.262,1.819 | 0.356 |
| Bisexual | 12.8 | 10.134 |  |  |  |  |  |  |  |  |  |  |  | 0.578 | 0.244,1.383 | 0.210 |

Notes: COR: crude odds ratio; AOR: adjusted odds ratio; Model 2: after controlling for psychoactive drugs use during sex; Model 3: after controlling for psychoactive drugs use during sex and HIV/STDs knowledge; Model 4: after controlling for psychoactive drugs use during sex, HIV/STDs knowledge, age stages of study, years in school, field of study, residence, sexual orientation and age of first sex.
